# Functional States of the Genome-Scale *Escherichia Coli* Transcriptional Regulatory System

**DOI:** 10.1371/journal.pcbi.1000403

**Published:** 2009-06-05

**Authors:** Erwin P. Gianchandani, Andrew R. Joyce, Bernhard Ø. Palsson, Jason A. Papin

**Affiliations:** 1 Department of Biomedical Engineering, University of Virginia, Charlottesville, Virginia, United States of America; 2 Department of Bioengineering, University of California San Diego, La Jolla, California, United States of America; Broad Institute of MIT and Harvard, United States of America

## Abstract

A transcriptional regulatory network (TRN) constitutes the collection of regulatory rules that link environmental cues to the transcription state of a cell's genome. We recently proposed a matrix formalism that quantitatively represents a system of such rules (a transcriptional regulatory system [TRS]) and allows systemic characterization of TRS properties. The matrix formalism not only allows the computation of the transcription state of the genome but also the fundamental characterization of the input-output mapping that it represents. Furthermore, a key advantage of this “pseudo-stoichiometric” matrix formalism is its ability to easily integrate with existing stoichiometric matrix representations of signaling and metabolic networks. Here we demonstrate for the first time how this matrix formalism is extendable to large-scale systems by applying it to the genome-scale *Escherichia coli* TRS. We analyze the fundamental subspaces of the regulatory network matrix (**R**) to describe intrinsic properties of the TRS. We further use Monte Carlo sampling to evaluate the *E. coli* transcription state across a subset of all possible environments, comparing our results to published gene expression data as validation. Finally, we present novel *in silico* findings for the *E. coli* TRS, including (1) a gene expression correlation matrix delineating functional motifs; (2) sets of gene ontologies for which regulatory rules governing gene transcription are poorly understood and which may direct further experimental characterization; and (3) the appearance of a distributed TRN structure, which is in stark contrast to the more hierarchical organization of metabolic networks.

## Author Summary

Cells are comprised of genomic information that encodes for proteins, the basic building blocks underlying all biological processes. A transcriptional regulatory system (TRS) connects a cell's environmental cues to its genome and in turn determines which genes are turned “on” in response to these cues. Consequently, TRSs control which proteins of an intracellular biochemical reaction network are present. These systems have been mathematically described, often through Boolean expressions that represent the activation or inhibition of gene transcription in response to various inputs. We recently developed a matrix formalism that extends these approaches and facilitates a quantitative representation of the Boolean logic underlying a TRS. We demonstrated on small-scale TRSs that this matrix representation is advantageous in that it facilitates the calculation of unique properties of a given TRS. Here we apply this matrix formalism to the genome-scale *Escherichia coli* TRS, demonstrating for the first time the predictive power of the approach at a large scale. We use the matrix-based model of *E. coli* transcriptional regulation to generate novel findings about the system, including new functional motifs; sets of genes whose regulation is poorly understood; and features of the TRS structure.

## Introduction

Complex regulatory networks control the transcription state of a genome and consequently the functional activity of a cell [1]. Even relatively simple unicellular organisms have evolved complicated networks of regulatory interactions, termed transcriptional regulatory networks (TRNs), to respond to environmental stimuli [1],[2]. External signals known to impact transcription in microorganisms include carbon source, amino acid, and electron acceptor availability, pH level, and heat and cold stress [2]–[6]. Mapping the links between these environmental growth conditions through signaling networks and ultimately to the resulting transcriptional response is of primary interest in the study of cellular systems [1]. Consequently, reconstructions of the TRNs of model organisms are underway [3].

To effectively describe the interconnected functions of the regulated genes and associated regulatory proteins within a given TRN, we recently developed a formalism involving a regulatory network matrix called **R** [7]. The **R** matrix represents the components (extracellular cues, metabolites, genes, and proteins, including regulatory activators and repressors) and reactions (regulatory rules) within a transcriptional regulatory system (TRS). We illustrated how, by using the fundamental properties of linear algebra, this matrix formalism allows characterization of TRS properties and facilitates *in silico* prediction of the transcription state of the genome under any specified set of environmental conditions.

Importantly, as previously reported (see [7]), the **R** matrix is distinct from existing approaches that use matrix formalisms and matrix algebra to analyze gene expression data (e.g., see [8]–[12]), as it describes relationships governing gene transcription derived from experiments characterizing how specific inputs regulate the expression of individual genes (e.g., ChIP-chip assays). In this way, the **R** matrix extends previous approaches for characterizing features of TRNs, including Boolean networks [2], [13]–[16], Bayesian networks [17], and stochastic equations [18] (see [1] for a review of the field). By representing the regulatory rules in matrix form, we can characterize the fundamental subspaces of the matrix (as described below), which in turn uniquely represent properties of the TRS that the **R** matrix contains. Furthermore, by using a “pseudo-stoichiometric” approach as discussed below, the **R** matrix representation of a TRN is consistent with, and thus easily integratable with, related approaches using stoichiometric matrices to computationally represent the reactions underlying metabolic and signaling networks [19]–[22].

To date, this approach for representing and analyzing TRSs has only been applied to relatively small systems, including the well-studied four-gene *lac* operon in *Escherichia coli* as well as a small 25-gene prototypic TRS [7]. Although these model systems have been useful for prototyping studies of the capabilities and behavior of the **R** matrix, a key unanswered question is how this approach scales to larger, more complex biological systems. Here we present first steps toward this end by assembling the **R** matrix for the genome-scale *E. coli* TRN, for which regulatory relationships have been previously characterized [23] and extensive experimental data (e.g., gene expression datasets) are available [3],[24]. To our knowledge, the work that we present here represents the first **R** matrix-based model of a genome-scale TRS, and this work has enabled us to gain important insights into the behavior of the **R** matrix at a larger scale, challenges associated with the scale-up, as well as the underlying biology of *E. coli* transcriptional regulation.

Specifically, we derived **R** directly from a previously developed genome-scale model of *E. coli* in which transcriptional regulatory rules were overlaid on a constraint-based model of metabolism [23]. This integrated transcriptional regulatory-metabolic model is well-suited for these initial genome-scale **R** matrix efforts as Boolean regulatory relationships are already defined and the behavior of this model has been well-studied using constraint-based analyses [23],[25]. To validate our **R** matrix analysis, we compared the expression states that we predicted for various environmental growth conditions with available gene expression data (as well as with predictions from the original Boolean model). We also explored the fundamental subspaces of a related matrix R* representing the complete *E. coli* TRS (to be defined below) to describe key systemic properties, including new hypotheses about network structure. Ultimately, this work yields an understanding of how the *E. coli* transcriptional regulatory program functions as a whole and demonstrates the utility of the regulatory network matrix formalism in studying transcriptional regulatory systems at the genome scale moving forward.

## Methods

We formulated a regulatory network matrix **R** for the genome-scale TRN of *E. coli*. Here, we summarize how we constructed the **R** matrix representing the *E. coli* TRN, sampled the space of possible environments for the TRN, and evaluated the fundamental subspaces of the matrix R* (for the complete *E. coli* TRS) to describe systemic properties.

### Updating an existing *E. coli* transcriptional regulatory network reconstruction

Significant efforts have focused on identifying the components and interactions that comprise the *E. coli* TRN [3]. These efforts have ranged from large-scale experimentation using post-genomic techniques [23],[26] to compiling previously reported regulatory relationships into literature-based representations of the *E. coli* TRN [5],[23]. Furthermore, several online resources have been developed to integrate both high-throughput as well as low-throughput (i.e., individual regulatory interactions elucidated through targeted experiments) experimental data into comprehensive databases [3],[24]. For example, EcoCyc [24] and RegulonDB [3] are two online resources that provide extensive information regarding transcription factor-target gene (DNA binding site) relationships. RegulonDB also catalogs known promoter sequences, experimentally-defined and computationally-predicted operons, as well as environmental stimulus-transcription factor relationships.

**Figure 1:**
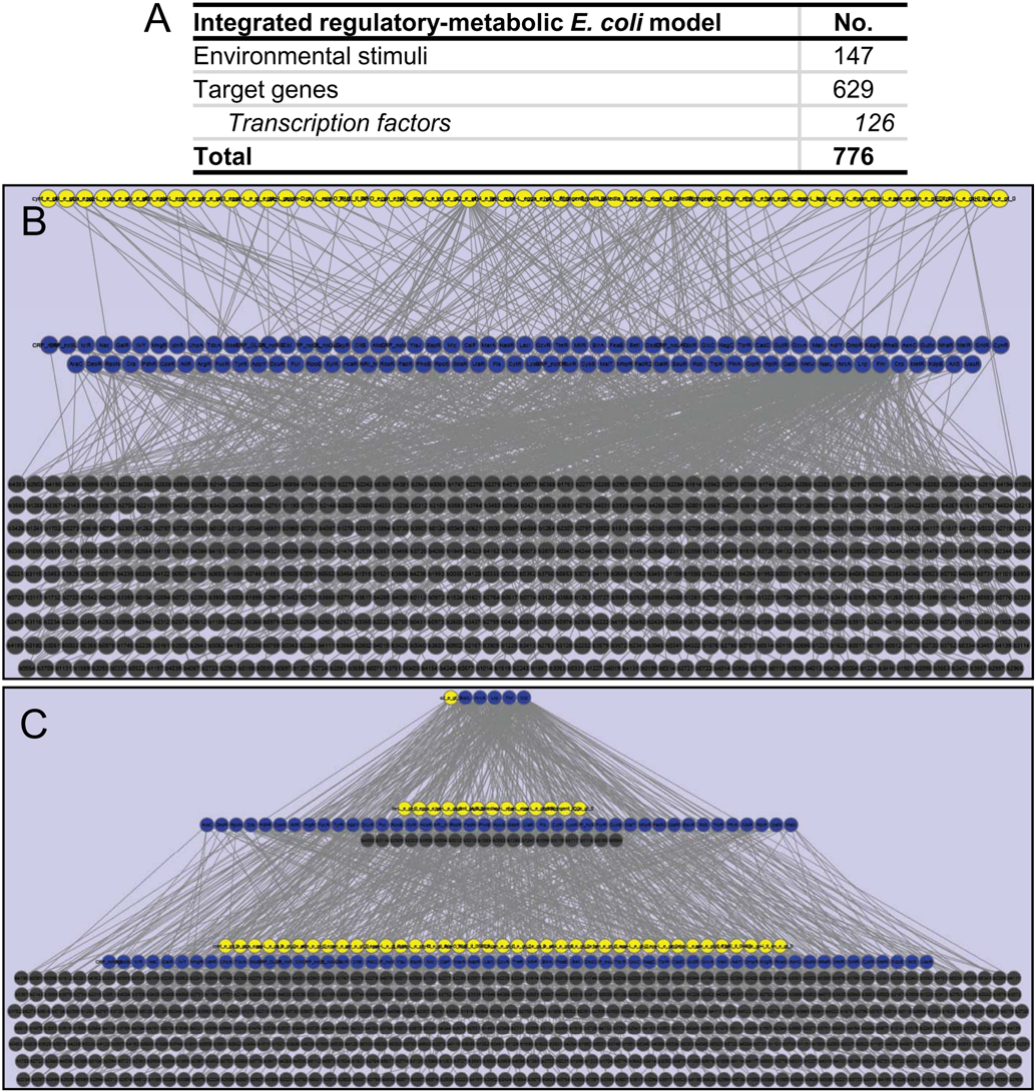
The *Escherichia coli* transcriptional regulatory system (TRS) at genome-scale. Panel A summarizes basic statistics of the *E. coli* transcriptional regulatory system (TRS). Panels B and C illustrate the components and their interactions in the *E. coli* TRS. Nodes constitute environmental cues (yellow), transcription factors (blue), and other target genes (dark gray). Edges (light gray) denote regulation (activation or repression) between nodes. As depicted in panel B, extracellular cues typically affect the expression of transcription factors, which in turn affect the expression of downstream target genes. In panel C, the hierarchical nature of the network is illustrated, with few global regulators affecting the transcription of many downstream genes. Nodes that have at least 25 connections appear in the top layer, those that have at least five but fewer than 25 connections are in the middle layer, and those that have fewer than five connections are in the bottom layer. As an example, the five nodes in the top layer are oxygen and the transcriptional dual regulators Crp, ArcA, Fnr, Lrp, and NarL. Path length seems to generally be a better indicator of broader regulatory impact as longer paths indicate more influence on other regulators and thus more regulatory targets. This network was visualized using Cytoscape [34].

These data formed the basis for a previous integrated regulatory-metabolic network reconstruction called iMC1010^v1^ [23]. In this model, Boolean rules dictating regulatory interactions were overlaid on a constraint-based model of *E. coli* metabolism. Here, these Boolean rules were used in the generation of a regulatory network matrix **R** for the genome-scale *E. coli* TRN. Three additional regulators (UlaR, MngR, and GntT) and their respective regulatory targets were added to the list of components and interactions, based on recent literature reports. In addition, several regulatory rules were either updated or refined to reflect current data, as measured using ChIP-chip assays and microarray experiments. The Boolean rules governing transcription of 46 new genes were added to the model, and the transcription rules for 11 other genes were modified. The underlying metabolic model was also updated from iJR904 [27] to the recently expanded *E. coli* model known as iAF1260 [28], including isozyme and multidomain subunit enzymes defined by similar Boolean relationships. The final *E. coli* TRN reconstruction was comprised of 147 environmental stimuli affecting 125 transcription factors that in turn influence 503 downstream target genes (see Figure 1 and Dataset S1). Ultimately, these target genes give rise to metabolic enzymes and transporters.

Importantly, constructing Boolean rules from experimental findings is not a trivial task. Published experimental data (ranging from high-throughput chip-ChIP assays or expression arrays spanning genome-scale information to “low-throughput” experiments focused on particular genes) are scoured for evidence indicative of a regulatory rule governing gene transcription, i.e., information describing how a transcription factor induces or represses transcription of target genes. As an example, the phrase “Crp induces the expression of *sdhC* within *E. coli*” is translated into a Boolean rule indicating that Crp is required for the transcription of the gene *sdhC* (succinate dehydrogenase subunit C) (i.e., “*sdhC*: **IF** (Crp)”). Conversely, a phrase that states “*sdhC* transcription is inhibited by either ArcA or Fnr” is translated into a Boolean statement “NOT(ArcA OR Fnr).” There are times when conflicts in the literature need to be resolved as well. In these instances, it is important to gauge which dataset appears to make a stronger case about a particular gene and its transcriptional requirements, in terms of the specific experimental conditions that were used and the corresponding likelihood for error. Alternatively, it may be possible to include both rules in the model separately and assess which one results in better model validation. The rules listed in Dataset S1 are accompanied by references.

### Generating a regulatory network matrix from Boolean rules

In order to define the regulatory network matrix **R** for *E. coli*, the Boolean rules from the updated integrated transcriptional regulatory-metabolic model for *E. coli* were translated into pseudo-stoichiometric relationships or “regulatory reactions.” As described in [7], the term “pseudo-stoichoimetric” is intended to indicate that this formalism delineates the relationships between components of the network (i.e., the chemical transformations) while not enforcing that the resultant reactions are mass-balanced in a strictly stoichiometric manner. Thus, effectively, the regulators (i.e., environmental cues and/or transcription factors that serve as inputs to a given gene regulatory rule/reaction) are “consumed” and the gene products or proteins (i.e., outputs of a gene regulatory rule/reaction) are “produced.” Importantly, however, this formalism can account for mass-balanced relationships as they become delineated in TRSs. To automate this conversion from Boolean logic to pseudo-stoichiometric reactions for large-scale systems, an expression parser was developed in Perl. Briefly, for each gene, the parser converted Boolean statements into regulatory reactions (i.e., pseudo-stoichiometric relationships) that could be represented in a **R** matrix. We used the formalism developed in [7] when implementing the expression parser to perform this conversion. For example, experimental data suggesting that transcription of (Gene 1) is induced if Metabolites A and B are both present within the system was represented in Boolean form, as in
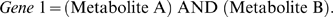
(1)


This Boolean rule was then converted by the parser into a reaction form, as in
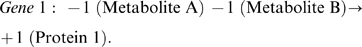
(2)


**Figure 2:**
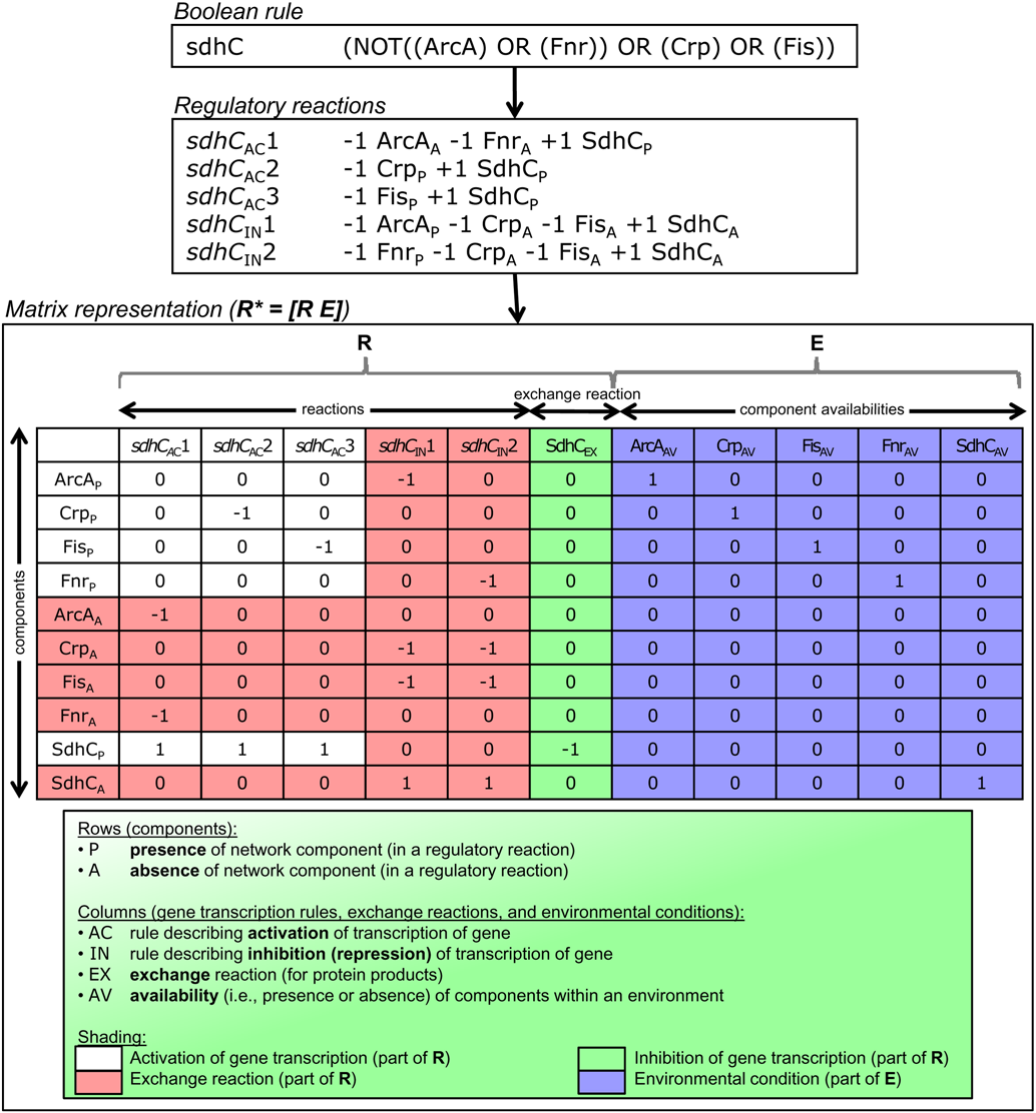
Generating a matrix representing the *E. coli* TRS from Boolean rules. The process for converting a Boolean rule describing gene transcription into a R matrix is illustrated using the gene encoding for succinate dehydrogenase membrane protein (*sdhC*) as an example. First, the Boolean rule is converted into a set of regulatory reactions, such that each clause of the Boolean rule corresponds to a single regulatory reaction. These regulatory reactions are then represented in matrix form (shaded in white), as described in [7]. In addition, to fully describe the system with R, reactions governing the repression of gene transcription for each gene (i.e., the converse reactions, shaded in red) as well as exchange reactions balancing the production of proteins (shaded in green) are added to R. Note that components delineating the presence and absence of regulators of *sdhC* are included within R, including ArcA_P_ and ArcA_A_, Crp_P_ and Crp_A_, Fis_P_ and Fis_A_, and Fnr_P_ and Fnr_A_. Finally, to evaluate the behavior of the regulatory model in the context of particular environments (i.e., sets of environmental cues that are present or absent), an environment matrix E describing component availabilities is placed adjacent to R, forming R* and capturing the complete TRS. The columns of E (shaded in blue) delineate the availability (i.e., presence or absence) of environmental cues, transcription factors, and proteins with respect to a particular environment. Multiple environments may be simulated by randomly selecting for the availability of environmental cues and other inputs. For complete details about the generation of R and R*, see [7].

When a Boolean rule was comprised of several clauses separated by “OR” statements, as in
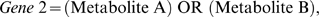
(3)the expression parser generated multiple regulatory reactions for the gene, as in
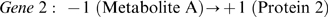
(4)and
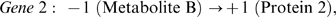
(5)as satisfying each clause (the presence of Metabolite A *or* the presence of Metabolite B) can result in protein synthesis independently. (The parser is included as Protocol S1.) Effectively, this parsing recast a gene's regulatory rule in disjunctive normal form (DNF) [29], with each clause of the DNF an independent regulatory reaction describing gene transcription. Importantly, the regulatory reactions distinguished the presence and absence of metabolites and transcription factors, as each of these regulates gene transcription differently. For example, consider a representative regulatory rule for the *E. coli* gene *sdhC*, shown at the top of Figure 2. Based on experimental data, *sdhC* is known to be transcribed if (1) both ArcA and Fnr are absent; (2) Crp is present; or (3) Fis is present. In other words, transcription of *sdhC* is induced by either Crp or Fis, and it is repressed by ArcA and Fnr in tandem. Consequently, the absence of ArcA (ArcA_A_ in Figure 2, where the subscripts “A” and “P” indicate absence and presence, respectively) as well as the absence of Fnr (Fnr_A_ in Figure 2) needs to be incorporated into **R**. In addition, fully describing the system with a **R** matrix required the inclusion of reactions governing both activation and repression of gene transcription for each gene as well as exchange reactions balancing the production of proteins (see an example of this for the gene *sdhC* in Figure 2); these effectively balanced the network so that functional states could be calculated as described below (i.e., an input was “consumed” and a product was “produced” without external manipulation). Reactions governing inactivation of gene transcription were included only for those genes whose protein products repress transcription of downstream genes. The compiled set of regulatory reactions effectively defined the *E. coli* **R** matrix, as illustrated in Figure 2. See Dataset S1 for a complete reaction listing. Ultimately, the complete **R** matrix was comprised of 1009 components (rows) spanning 1685 reactions (columns), including 579 exchange reactions. This study thus constituted the construction of the first genome-scale **R** matrix for an organism. The **R** matrix is unique among matrix-based approaches in the field of transcriptional regulation in that it catalogs experimentally-characterized relationships governing gene transcription, thereby facilitating *in silico* expression state analysis. Other matrix analyses have interrogated experimental gene expression data (see [8]–[12] for examples of these studies) without necessarily having an underlying functional and/or predictive model.

### The environment matrix and randomly simulating environments

To evaluate the behavior of the genome-scale *E. coli* TRS in the context of particular environments (i.e., sets of environmental cues defined as present or absent), we further defined environment matrices. Each environment matrix, **E**, was comprised of the same number of rows as **R**. The columns of **E** delineated the availability (i.e., presence or absence) of environmental cues, transcription factors, and proteins with respect to a particular environment. Consequently, in the case of the *E. coli* TRN, there were 776 different columns in **E**, one for each unique metabolite, transcription factor, or target gene. For a given environment, **E** is appended to **R** to form R*, which captures the complete TRS (see Figure 2 for an example of how a particular gene rule was combined with a representative environment to yield R*, as well as [7] for further details about this process). In this way, multiple environments were simulated by randomly selecting for the availability of environmental cues and other inputs (see below). These environments were used to assess the behavior of the system across a random sampling of all possible environments. See Dataset S2 for a listing of 1000 randomly-sampled environments (as introduced below). In addition, separately, we evaluated two specific environments (anaerobic and aerobic minimal media) for which gene expression data have previously been experimentally characterized, as described below (see Dataset S3 for these environments).

#### Computing expression profiles

**Figure 3:**
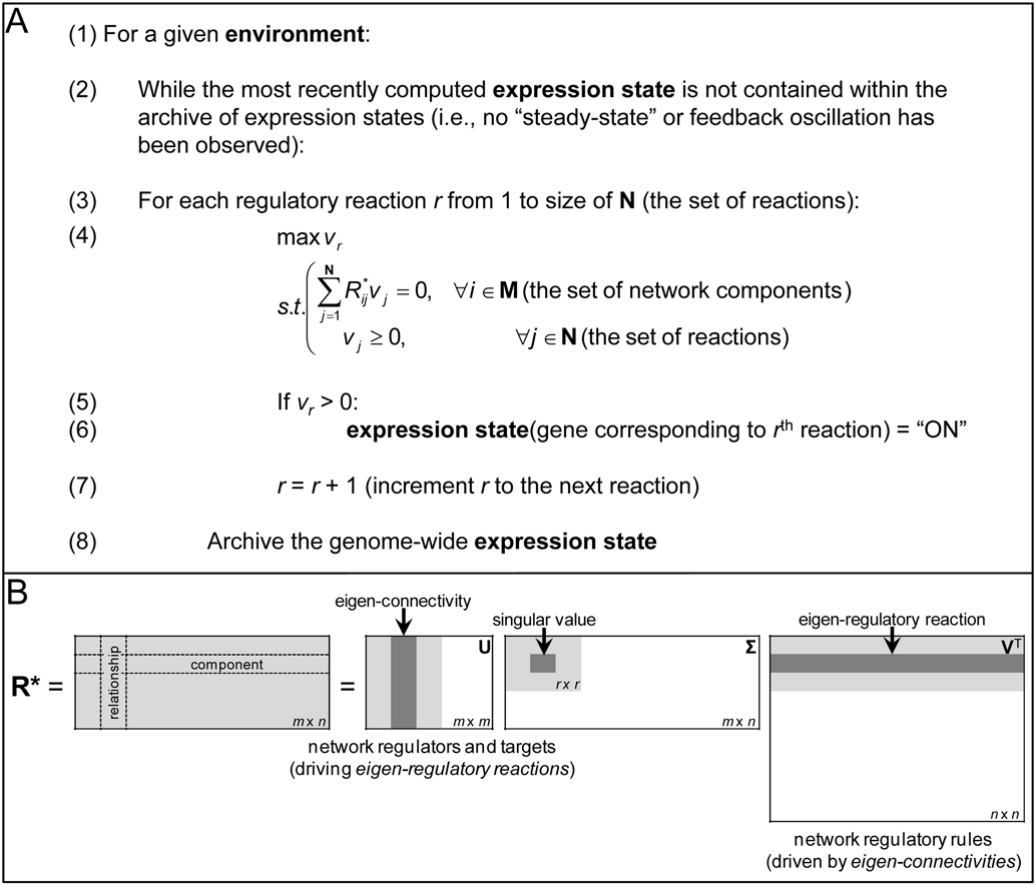
Key analysis techniques to interrogate the regulatory network matrix. Panel A depicts the linear programming (LP) problem that we solve in order to predict which genes are turned “on” (or “off”) in response to a given environment. In particular, for a given environment (line 1), we iterate through each regulatory reaction (lines 3 and 7) one by one, optimizing it while enforcing that the components are balanced and all reactions within the system proceed in the forward direction (line 4). Note that M is the set of network components and N is the set of regulatory reactions governing (activation and repression of) gene transcription. If the flux of the *r* ^th^ reaction being optimized is positive, then we consider that reaction to be “active” and the corresponding gene to be “on” (lines 5–6). We repeat this process until the expression state of all genes matches a prior expression state (lines 2 and 8), suggesting that a steady-state has been attained or the expression states demonstrate oscillatory behavior characteristic of one or more feedback loops. The expression state at this point is the predicted expression state corresponding to the environment (E) contained within R*. Panel B illustrates how a given R* matrix is decomposed into U, Σ, and V matrices using singular value decomposition (SVD). We further depict how “eigen-connectivities” describing network regulators and targets are contained in the column and left null spaces (within U), whereas collections of regulatory rules driven by eigen-connectivities (or “eigen-regulatory reactions”) are contained within the row and null spaces (within V^T^). (See Text S2 for additional details.)

Although certain fundamental subspaces of R* ( = [**R E**]) describe the expression state of the system in the context of a particular environment (see below), we utilized a linear programming (LP) strategy to efficiently predict a route through the network given an environment, i.e., an expression profile (see Figure 3A). This approach is similar to flux-balance analysis (FBA), which has been used extensively in metabolic systems to predict the rates of network reactions [30]. Briefly, given a stoichiometric network reconstruction of a metabolic system and assuming steady-state conditions, FBA is a constraints-based approach that optimizes for a particular flux, all the while ensuring that certain biological constraints such as mass balance and thermodynamics are maintained [31].

Here, we initially assumed that each gene product was absent from the system and constrained all 1106 regulatory relationships (or pseudo-stoichiometric reactions) in the forward direction. We used our LP strategy to iterate through the regulatory reactions contained within R*, optimizing the “flux” associated with each reaction (Figure 3A, line 4). If we observed that we were able to obtain a flux distribution with a nonzero flux through the “optimized” reaction, then we predicted that the corresponding reaction is “active” and associated gene “expressed.” This LP strategy is predicated on a balance on each network component; the consumption of an input as part of a regulatory reaction is “balanced” by the presence of the input within the environment, as represented in **E**, and only if the input is present can the associated gene be “expressed.” To account for the different hierarchical layers of control in transcriptional regulation, this process of optimizing for the flux through each regulatory reaction was repeated multiple times until the gene expression predictions (i.e., “expressed” or “not expressed”) were consistent for all the genes within the model across multiple iterations (i.e., a “steady-state” was achieved) or until an oscillation characteristic of one or more feedback loops was observed in the regulatory relationships. Notably, we varied the order in which we optimized the regulatory reactions and ran our optimization procedure, and the results were consistent, suggesting that we achieve “global” steady-states (data not shown). (See Figure 3A and the accompanying figure caption for additional details about our optimization procedure.) As the matrix formalism is applied to higher-order regulatory systems in the future, this issue of “global stability” will be further explored. We also considered alternate objective functions, such as simultaneously optimizing for all the regulatory reactions within the TRN (see Text S1 for details).

Our LP strategy for predicting an expression state for a TRS in response to a given environment is different from regulated flux balance analysis (rFBA), which has been proposed for the analysis of an integrated regulatory-metabolic network (see [6]). rFBA incorporates gene expression predictions (as computed by a Boolean or other model of transcriptional regulation) into the FBA constraints for a metabolic network. For example, the metabolic fluxes for reactions corresponding to genes that are not expressed (according to a Boolean model of regulation) are constrained to zero units in rFBA. Our LP strategy instead determines which genes are transcribed through a TRS in response to a given environment.

### The fundamental subspaces of R*

We analyzed the fundamental subspaces of the regulatory network matrix to describe properties of the *E. coli* TRS. Specifically, a given TRN represented by **R** responds to environmental signals whose states (i.e., presence or absence) need to be specified [7]. Consequently, the **R** matrix is further combined with an environment matrix **E** that characterizes the environment against which a set of regulatory rules is to be evaluated [7]. As the combination of **R** and **E** (i.e., the matrix R*) captures the TRS being analyzed, we interrogated the fundamental subspaces of this matrix to describe properties of the *E. coli* TRS.

**Figure 4:**
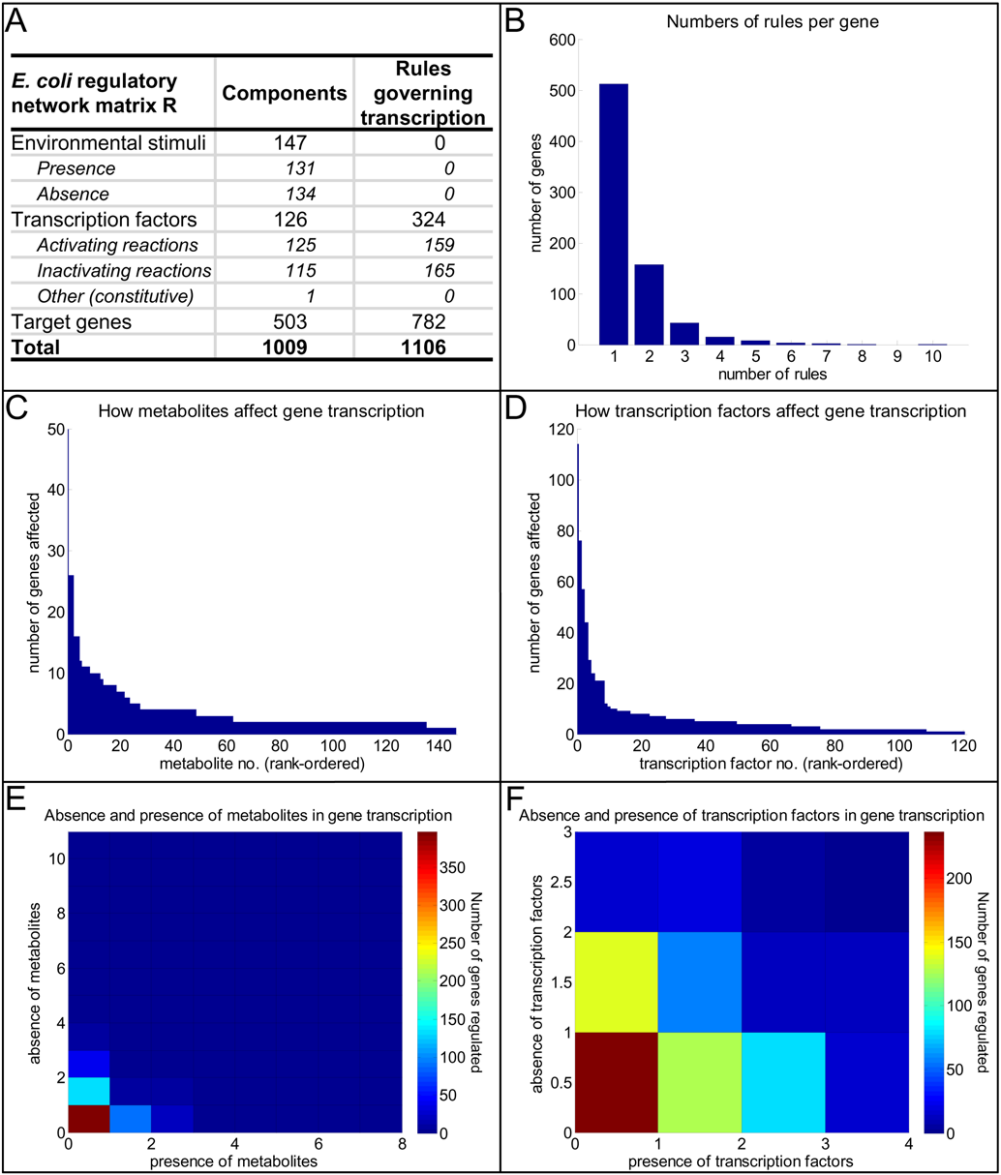
Characteristics of the regulatory network matrix for *E. coli*. Panel A summarizes basic statistics of the regulatory network matrix R. Panel B shows the number of rules (i.e., regulatory reactions) per gene. Panels C and D depict the numbers of genes that each metabolite and transcription factor, respectively, affect. The metabolites and transcription factors along the *x* axes are rank-ordered according to the numbers of genes that they affect. Panel E illustrates the numbers of genes requiring the presence or absence of the corresponding numbers of metabolites. Panel F illustrates the numbers of genes requiring the presence or absence of the corresponding numbers of transcription factors.

Briefly, the four fundamental subspaces of a matrix, namely the column space, left null space, row space, and null space, describe key properties of the matrix and, in turn, the system that the matrix represents [32]. In the case of R*, these fundamental subspaces were previously shown to represent key system properties for a prototypic TRS as well as the *E. coli lac* operon TRS [7]. As shown in Figure 3B and described in more detail in Text S2, singular value decomposition (SVD) is used to decompose a matrix into three matrices, often named **U**, **Σ**, and **V** (see Figure 3B) [32], and these matrices delineate the four fundamental subspaces of the original matrix (see [7] and Figure 4B). We performed SVD to characterize the fundamental subspaces of multiple R* matrices (describing different randomly-generated environments) for the *E. coli* TRS, and we summarize the results below. As we describe in our “Results” below, our understanding of the four fundamental subspaces of R*, which we originally proposed in [7] on the basis of our work with two small-scale systems, has been considerably enhanced by the extension of **R** and R* to the genome-scale *E. coli* TRS.

Besides the fundamental subspaces, we also computed the angles between columns and rows of R* as these are also informative about the TRS that R* represents. For every pair of column (or row) vectors contained in the matrix, we computed the angle between the vectors by taking the inverse cosine of the dot product between the vectors. The angles between columns of R* indicate the similarity or dissimilarity in the rules governing regulation of the genes. For example, a small angle between a pair of columns suggests that the regulatory rules of the two corresponding genes are relatively similar and affect the state of the TRS in a similar fashion. Likewise, angles between rows of R* indicate the overall similarity or dissimilarity of network component participation in the generation of expression states. For instance, a large angle between a pair of rows (e.g., extracellular cues) suggests that the two network components are relatively dissimilar and affect the transcription of different sets of genes or affect the transcription of the same genes in different ways (e.g., one might be a transcriptional activator while the other is a repressor).

### Implementation details

As described above, a parser that converts Boolean logic into regulatory reactions was implemented in Perl. A freely available extreme pathway analysis program (ExPa, University of California, San Diego) [33] was used to convert the regulatory reactions into a regulatory network matrix. Ultimately, this matrix was imported into MATLAB v. 7.6 (part of the R2008a release package, MathWorks, Natick, MA), and code was written to explore the structure of the matrix and to simulate the behavior of the TRN under various environments. The MATLAB representation of the *E. coli* **R** matrix and a sample R* matrix is provided in Protocol S2. Maps of the *E. coli* TRS were constructed using Cytoscape v. 2.6 [34].

## Results

Here we present initial steps toward applying the regulatory network matrix formalism to the genome-scale *E. coli* TRN. In order to facilitate this process, a previously developed model of the *E. coli* TRN [23] was updated to reflect recently published regulatory interactions as well as an expansion of the underlying metabolic model [28] (see Dataset S1). The resulting updated Boolean rules describing the regulation of the underlying components were then used to generate pseudo-stoichiometric relationships or “regulatory reactions.” The compilation of these reactions represents the scope of the **R** matrix for *E. coli* and illustrates the complexity involved when applying this approach to a genome-scale system.

### Characteristics of the *E. coli* TRS

The **R** matrix of the *E. coli* TRS is comprised of 1009 components (rows) spanning 1685 reactions (columns), including 579 exchange reactions (see Figure 4A). As illustrated in Figure 1C, the *E. coli* TRS exhibits a hierarchical structure, as highly connected global regulators act broadly to influence the expression of major and minor regulators and thus directly and indirectly affect the transcription of numerous target genes. Examples of global regulators include traditional regulators such as transcriptional dual regulator Crp, which senses cyclic AMP (cAMP) levels and thus monitors the nutritional status of the cell, and nucleoid binding proteins such as histone-like nucleoid structuring protein (H-NS) and factor for inversion stimulation (Fis), which bind the chromosome and thus influence its topology within the cell in addition to directly impacting gene expression. Alternative sigma factors such as RNA polymerase sigma factor (RpoS) are also found among this class of proteins as they influence the expression of diverse and numerous targets in response to various cellular stresses.

#### Scope of the genome-scale R matrix

As detailed in [7], in order to properly account for genes that are regulated by the absence of regulatory factors, both the presence and absence of individual components had to be accounted. For instance, as illustrated in Figure 2, both ArcA_P_ (the presence of transcriptional dual regulator ArcA) and ArcA_A_ (the absence of ArcA) are components of the **R** matrix representation of the TRN. This convention approximately doubles the number of environmental stimuli and transcription factors included. Since certain regulators respond to multiple factors, the numbers of rules are more than the numbers of genes. Figure 4A summarizes the scope of the **R** matrix for the genome-scale *E. coli* TRN.

#### Features of the R matrix

Within the **R** matrix describing the *E. coli* TRN, the number of regulatory reactions (or pseudo-stoichiometric relationships) is of the same order of magnitude as the number of regulated genes. In other words, most genes within the TRN are described by only a few independent regulatory reactions (or columns within **R**) (see Figure 4B). Only 15 genes have five or more independent rules or regulatory events. Interestingly, several of these genes are themselves transcriptional activators or repressors, including transcriptional dual regulators *glcC* (5 inputs) and *marA* (5 inputs), transcriptional activators *tdcA* (10 inputs) and *feaR* (8 inputs), transcriptional repressor *exuR* (5 inputs), and phenylacetaldehyde dehydrogenase (*feaB*, 5 inputs). Consequently, the number of reactions represented in **R** is only modestly increased over the number of regulated components themselves, and the numbers of genes and regulatory reactions are on the same order of magnitude (743 genes versus 1106 independent regulatory reactions).

The transcription of most genes is governed by only a few regulators (Figures 4C, 4D, 4E, and 4F). In particular, most metabolites affect the transcription of fewer than 10 genes (Figure 4C), and the majority of transcription factors regulate less than 10 downstream target genes (Figure 4D). Some notable exceptions include oxygen, D-glucose, and the amino acid leucine, which affect 50, 26, and 26 genes, respectively. As oxygen and glucose are essential for cell survival, it is perhaps obvious that they dominate the expression of many more genes than other environmental cues. In addition, the most pervasive transcription factor is Crp, which regulates 114 downstream genes. Other key regulating proteins include the well-studied transcriptional dual regulators Fnr (which affects 76 genes), ArcA (57 genes), and NarL (44 genes); the transcriptional repressor PurR (24 genes); RNA polymerase sigma N factor (RpoN, 21 genes); RpoS (21 genes); and transcriptional dual regulator CysB (21 genes). Data in Figures 4E and 4F demonstrate how the majority of genes are regulated by the presence (along the *y* axis) or absence (along the *x* axis) of few metabolites and transcription factors, respectively. Notable exceptions include *exuR*, which requires 8 different environmental cues to be absent for transcription.

### Expression states

#### Model validation

**Figure 5:**
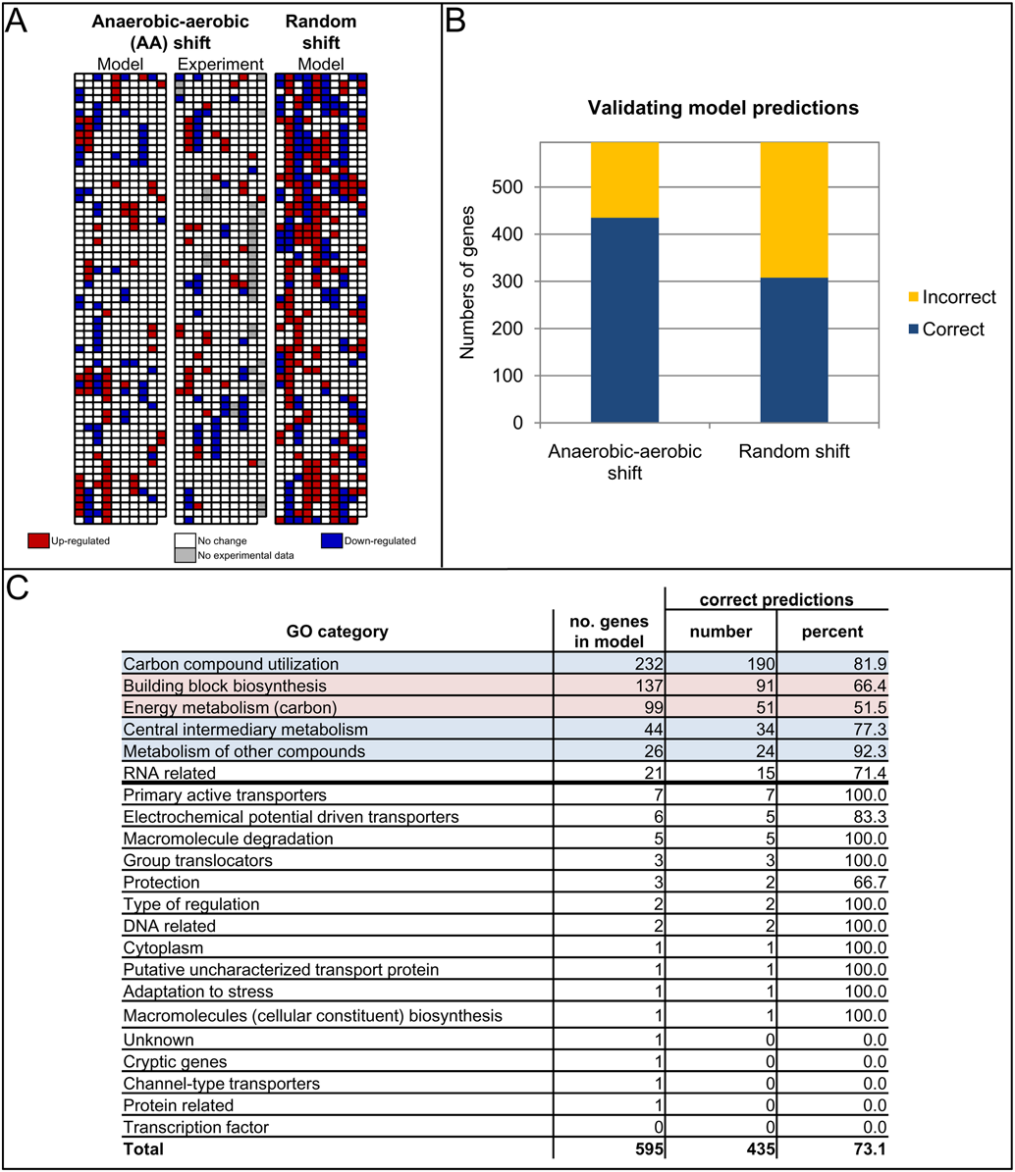
Model validation. Panel A compares side-by-side the *in silico* and *in vitro* expression profiles for the anaerobic-aerobic minimal media shift in *E. coli*. Genes whose expression level decreases from the anaerobic to aerobic medium are shaded in blue, whereas genes that are up-regulated or unchanged are shaded in red and white, respectively. See Dataset S4 for a legend describing the placement of the genes within this panel. In general, there is strong concordance between the *in silico* and *in vitro* data sets for the directed studies. However, a random shift simulated *in silico* and also depicted illustrates poor concordance with experimental data as a random control. Panel B depicts the accuracy between the *in silico* and *in vitro* expression profiles for the two simulated shifts. Panel C breaks down the model validation by gene ontology (GO) category. Specifically, for the anaerobic-aerobic minimal media shift, presented are the percentages of genes within each GO category for which the predictions from our model matched those from the experiments. GO categories with large gene populations that exhibit strong (and poor) validation are shaded blue (red) for emphasis.

To validate the *in silico* model of *E. coli* transcriptional regulation as described in the **R** matrix, we generated expression profiles of the 629 regulated genes in response to two distinct environments for which gene expression data have previously been measured [23]. Specifically, these environments constituted anaerobic and aerobic minimal media conditions, as detailed in [13] and summarized in Dataset S3. We used a linear programming approach tailored specifically to R* as described above (see “Methods”) to determine the expression states for the two environments. We then compared the differential expression owing to the anaerobic to aerobic shift (i.e., which genes went from being turned “off” in response to the anaerobic minimal media to being turned “on” in response to the aerobic minimal media, which ones went from “on” to “off,” and which ones were unchanged) between our *in silico* predictions and actual experimental data taken from [23]. The results of this validation are presented in Figures 5A and 5B (see Dataset S4 for a legend defining the genes shown in Figure 5A). The differential expression for the anaerobic-aerobic shift predicted by the R* matrix-based analysis demonstrated 73 percent agreement with the experimentally-characterized profile, i.e., the change in expression between the anaerobic and aerobic conditions was consistent between the R* matrix predictions and the expression data for 73 percent of the genes contained within the matrix. As a control, we compared the differential expression that the model predicted between two randomly-generated environments with that measured experimentally for the anaerobic-aerobic shift, and the accuracy in this case was only 51 percent (see Figures 5A and 5B), or significantly less than the 73 percent when corresponding conditions were paired (*p*-value<0.01). This result was important because it emphasized that our validation was not purely an artifact of the regulatory rules or our LP analysis of the R* matrix. We also compared the expression states predicted by our R* matrix analysis for the anaerobic and aerobic minimal media with those predicted by an equivalent Boolean model for the same environments, and we obtained 100 percent agreement (results not shown), thus ensuring that our representation of a Boolean TRS in a pseudo-stoichiometric matrix form does not introduce any sources of error.

We further analyzed the agreements and disagreements between predicted and observed expression profiles for the anaerobic-aerobic shift by gene ontology (GO) categories (see Figure 5C). Specifically, we computed the percentages of genes within each GO category for which the model predictions matched with the experiments. This analysis enabled us to identify specific GO categories containing large numbers of genes (>20 genes) but yet exhibiting less than 70 percent validation, including energy metabolism and building block biosynthesis-related genes (see GO categories shaded in light red in Figure 5C). These GO categories represent starting points for further experimental characterization of regulatory relationships within the *E. coli* TRS. By contrast, certain GO categories containing large numbers of genes were very well validated (see GO categories shaded in light blue in Figure 5C). We discuss this result below (see “Discussion”).

#### Sampling functional states

**Figure 6:**
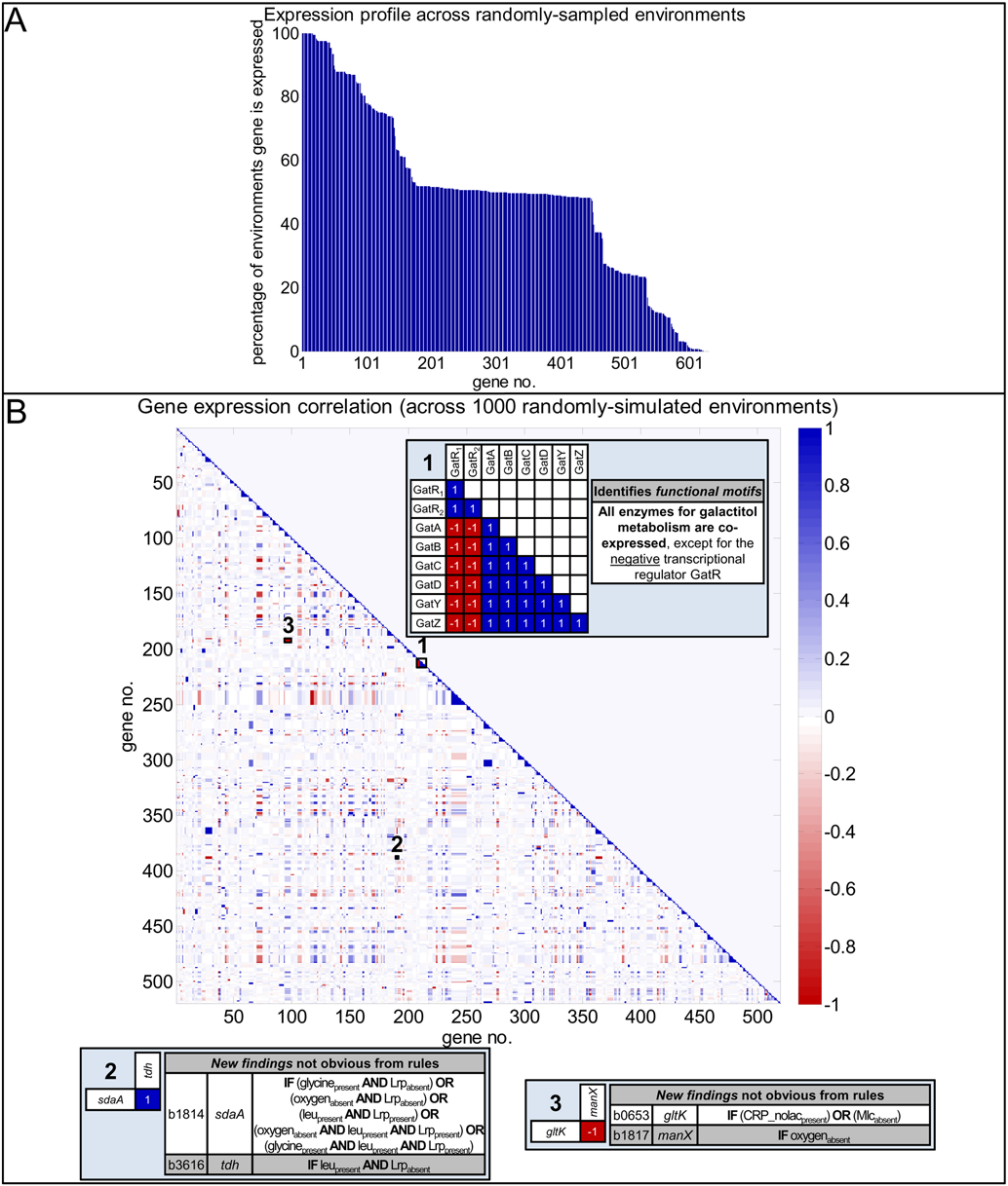
Expression states. Panel A presents the genes as a (rank-ordered) function of the percentage of 1000 randomly-simulated environments in which they are expressed. The majority of genes are expressed in a fraction of the environments, although some genes are expressed ubiquitously and others never. See the text as well as Dataset S5 for a discussion of these relationships. Panel B depicts an expression correlation matrix, delineating the level of correlation between pairs of genes across the 1000 randomly-simulated environments. Colors indicate that the expression of two genes is correlated (blue if the expression of one gene is correlated with that of another gene, and red if the expression of one gene is correlated with the lack of expression of another gene (or vice-versa)), and the darker the color the stronger the correlation observed. Note that genes that are always expressed or never expressed across the 1000 environments are excluded from this analysis. Examples of interesting insights gained from the gene expression correlation matrix are highlighted.

We further investigated functional states of the *E. coli* TRS by generating expression profiles for each of the 629 regulated genes, including 125 transcription factors, across 1000 randomly-sampled environments. In other words, effectively we generated *in silico* “microarrays” for each of the 1000 randomly-sampled environments. The 629 regulated genes are plotted in Figure 6A as a (rank-ordered) function of the percentage of these 1000 environments in which they are expressed. While the majority of genes were expressed in a fraction of the environments, 14 were expressed in all of the simulated environments and seven were expressed in none of the simulated environments. By contrast, 176 genes were expressed in between 49 and 51 percent of the simulated environments. We explored these genes (see Dataset S5) further. Specifically, the set of genes that were ubiquitously expressed across the randomly-sampled environments is important for carbon source uptake and energy metabolism, and thus likely essential for *E. coli* survival. However, the specific genes contained within this set are not obvious. For example, the large and small subunits of glutamate synthase (*gltB* and *gltD*, respectively) are expressed across all 1000 simulated environments, even when alternate carbon sources are supplied within the environmental medium. This result could be suggestive of incorrect or incomplete gene annotations, or of novel pathways in which these genes participate. Likewise, unexpectedly, none of the 125 transcription factors included within the reconstructed *E. coli* TRN was ubiquitously expressed; rather, many operon-specific transcription factors such as *cytR*, *gcvR*, *ilvY*, *kdpE*, *uhpA*, and *uhpB* were expressed in only about half of the simulated environments. By contrast, the genes that were never expressed included those for which redundant processes exist within *E. coli*, including for example dicarboxylate DAACS transporter (*dctA*) and dicarboxylate Dcu transporter (*dcuB*). There is evidence within the literature that four dicarboxylate transporters exist within *E. coli* and that *dctA* and *dcuB* are only expressed if the other two are not [35]. Importantly, these types of outliers are not immediately obvious by simply examining the Boolean rules governing gene transcription. For example, in the case of the gene *dctA*, the regulatory reaction is
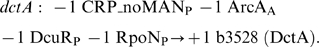
(6)


Inspecting this reaction by itself does not immediately suggest that *dctA* would be expressed in zero of the simulated environments, particularly since multiple key transcription factors, including Crp (in the absence of mannose), ArcA, transcriptional activator DcuR, and RpoN, are involved. However, the nature of upstream regulatory interactions is such that the precise combination in which these transcription factors need to be present for *dctA* to be transcribed is exceptionally rare. Consequently, this redundancy that is apparent within the *E. coli* regulatory network is not easy to infer without the type of quantitative analysis afforded by the regulatory network matrix.

#### Correlated gene sets

Our analysis of *E. coli* expression states across 1000 random environments also enabled the generation of a gene expression correlation matrix (shown in Figure 6B) containing the level of expression correlation across the environments for every pair of genes within the *E. coli* TRS. In particular, the correlation coefficient (*r_ij_*) describing the level of expression correlation between every pair of genes *i* and *j* within the TRS was computed. Pairs of genes that are consistently expressed together (either consistently “on” and/or “off” together) have positive correlation coefficients (and are shaded in blue), whereas pairs of genes in which one gene is consistently expressed while the other is not (and vice-versa) have negative correlation coefficients (and are shaded in red). When the expression between a pair of genes is completely inconsistent, the *r_ij_* value is equal to zero (and the intersection of the genes within the matrix is shaded in white).

Correlated gene expression is an indication of structural motifs of the TRN, such as operons (as illustrated by the galactitol PTS permease *gat* operon (item “1” in Figure 6B)). Indeed, as genes belonging to an operon are often found adjacent to one another within the genome (and consequently appear as such within the reconstructed *E. coli* TRS), many operons are easily found along the diagonal of the matrix as evidenced by the striking blue (indicating strongly correlated expression) that appears there. In addition, novel insights not necessarily obvious from a simple inspection of the regulatory rules were attained, as in the case of the genes *L*-serine deaminase I (*sdaA*) and threonine dehydrogenase subunit (*tdh*) (marked as “2” in Figure 6B). Based on literature that was used to construct the **R** matrix describing the *E.* coli TRS, *sdaA* is transcribed if one of several rules are satisfied, as listed in Figure 6B, whereas *tdh* is transcribed if leucine is present but Lrp is absent (see “2” in Figure 6B). By simple inspection, these rules would not necessarily suggest whether *sdaA* and *tdh* would be expressed together. However, our functional state analysis revealed that in fact the expression of *sdaA* and *tdh* was correlated 100 percent of the time. The rules governing the expression of *sdaA* and *tdh* are comprised of transcription factors whose expression themselves are governed by independent rules, such that there exists an interconnectivity between *sdaA* and *tdh*. This result is indicative of the complexity that exists within the *E. coli* TRS and the interdependency of the TRN, and perhaps even suggestive of evolutionary forces that have selected for physiology such that different input requirements for the transcription of these genes ultimately yield the same outcome for a given environment. Such correlation may also be suggestive of pharmacological strategies as inhibiting the function of one of these gene products may effectively target functions related to the other. Likewise, from a biological standpoint, it is interesting that the genes *gltK* and *manX* are always expressed opposite of one another (see “3” in Figure 6B). This result is not obvious by simply inspecting the Boolean rules that govern the transcription of these genes (again, see “3” in Figure 6B). However, as *gltK* is an integral membrane component of the glutamate ABC transporter and *manX* is a mannose PTS permease, this result suggests that *E. coli* elicits a different transcriptional regulatory program in response to these two different sugars. Furthermore, it provides evidence of how the prokaryote has evolved direct, specific responses so that only those genes necessary for a given environment are actually transcribed, thus conserving energy.

The analysis of correlated gene sets within the *E. coli* TRS, made easier by the regulatory network matrix formalism and associated analysis, thus enables novel insights about structural and functional properties of the system to be hypothesized, enhancing our understanding of basic biology and potentially suggesting strategies for therapeutic development. (See Text S3, Figure S1, and Dataset S6 for the gene expression correlation clusters.)

### Fundamental subspaces of R*

To further evaluate properties of the *E. coli* TRS, we considered fundamental subspaces of multiple R*, with each R* corresponding to a unique, randomly-generated environment. A representative subset of these randomly-generated environments is presented in Dataset S2. We performed singular value decomposition (SVD) on each R*, as described in [7] and shown in Figure 3B, yielding R* = **U•Σ•V** ^T^. The diagonal entries of the matrix **Σ** = diag(*σ*_1_, *σ*_2_, … , *σ_r_*), where *r* is the rank of R* and *σ*_1_≥*σ*_2_≥…≥*σ_r_*, indicate the relative contribution of the corresponding left singular vector (a column of **U**) and right singular vector (a row of **V** ^T^) in the overall construction of the TRS [36]. Note that an important feature of SVD is that the singular vectors are orthonormal to each other and consequently each principal mode is decoupled from all the others.

**Figure 7:**
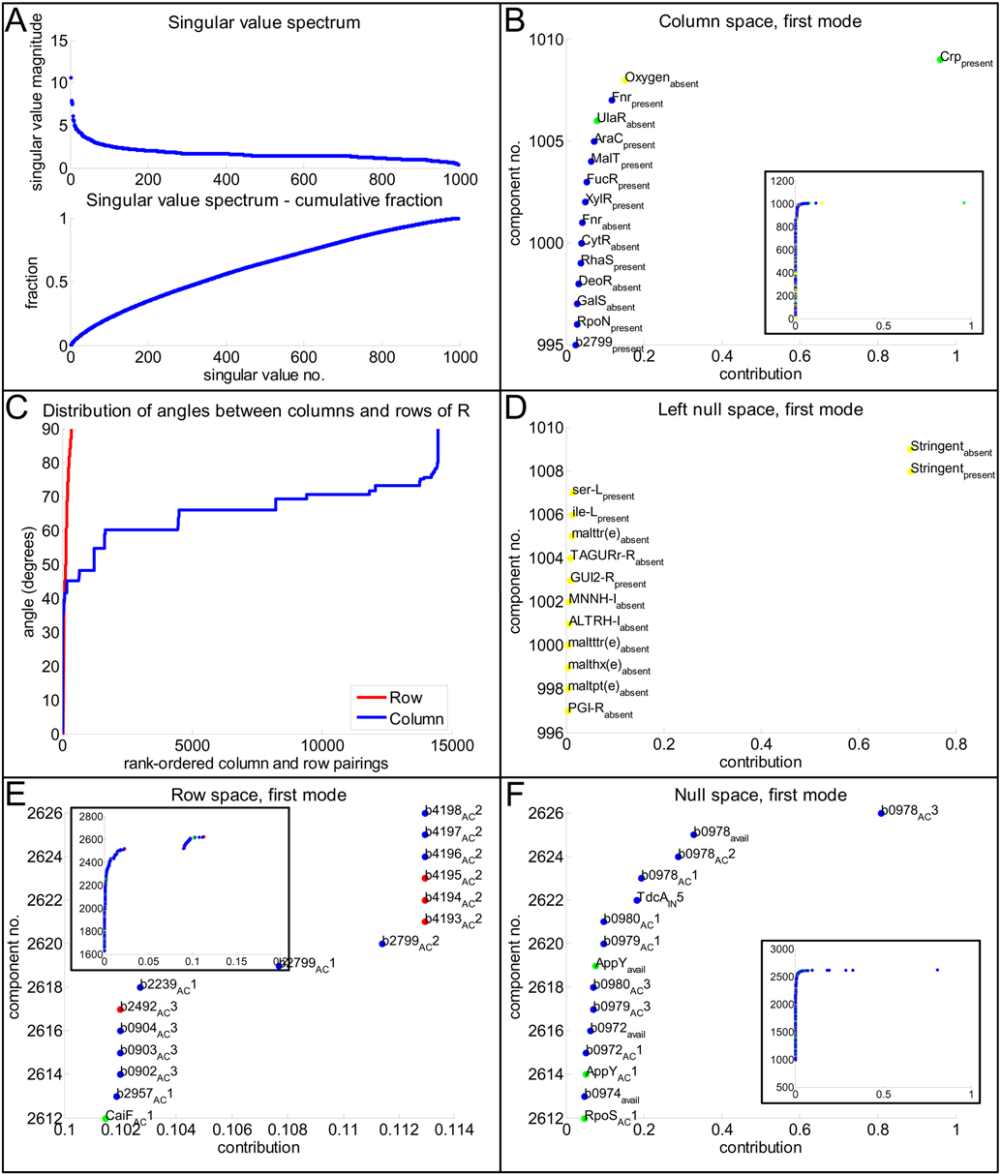
Fundamental subspaces. The results of singular value decomposition (SVD) of a representative *E. coli* transcriptional regulatory system (TRS) matrix R* are presented. Panel A illustrates the rank-ordered singular values of the matrix (top) and the corresponding cumulative sum of the singular values (bottom). Panels B, D, E, and F depict the first modes of the column space, left null space, row space, and null space, respectively, of R*. In panels B, E, and F, the 15 nodes (components or reactions) that contribute most to the corresponding subspace are presented in the larger figure. The insets of these panels illustrate the complete modes, and here nodes are shaded according to Gene Ontology (GO) classifications: yellow dots correspond to extracellular metabolites; yellow crosses correspond to transcriptional activators and repressors; cyan corresponds to periplasm and surface genes and proteins; blue corresponds to metabolic genes and proteins; green corresponds to regulatory genes and proteins; red corresponds to transport genes and proteins; black corresponds to genes and proteins of unknown (putative hypothetical) function; and magenta corresponds to genes and proteins belonging to other categories. The column and left null spaces are comprised of network components, including the presence and absence of components, while the row and null spaces are comprised of regulatory reactions (rules), including environmental availabilities of metabolites and regulated protein products. Panel C illustrates the angles between pair-wise combinations of rows (red) and columns (blue) of the *E. coli* R matrix.

Interestingly, across many different randomly-generated environments, the singular value spectra of the matrix R* representing the genome-scale *E. coli* TRS (i.e., the singular values *σ*_1_, *σ*_2_, … , *σ_r_*) were relatively consistent, suggesting that the environment does not contribute significantly to the properties of the TRS. The number of inputs to the system ( = 147 environmental cues) constitutes less than six percent of the columns within R*. Furthermore, although the singular value of the first mode is larger than that of the next closest mode (10.60>7.895), the singular value spectrum of a given R* is rather uniformly distributed, as shown in a representative spectrum in Figure 7A. In other words, the information content of a given R* is evenly distributed throughout the matrix, or, alternatively, there are few components or reactions that dominate the genome-scale *E. coli* TRS that R* describes. This result is particularly insightful as it contrasts with the structural hierarchy (with few regulators affecting many genes, and many affecting few genes) that is evident by purely inspecting the network map (Figure 1C). Furthermore, whereas in metabolic networks approximately 27 percent of the information content of a stoichiometric matrix is often captured in the first four principal modes (less than one percent of all the principal modes) [36], here, to capture an equivalent information content of a regulatory matrix, the first 150 principal modes (or about 15 percent of all the principal modes) must be recapitulated. Thus, although a simple inspection of the network would suggest that only a small handful of regulators control a large fraction of the network, control of the TRN is significantly more distributed. We discuss this result in detail below (see “Discussion”).

#### Column space

The column space is spanned by the first *r* left singular vectors in **U** [32]. For metabolic systems, each mode in the column space has previously been labeled an “eigen-reaction,” describing principal chemical transformations of the metabolic network. For example, the dominant eigen-reactions for metabolic systems have been comprised of transitions of cofactors participating in energy, redox, and phosphate metabolism, including the conversion of ATP to ADP and P*_i_* [36]. The first mode of the column space for the R* matrix describing the *E. coli* TRS is illustrated in Figure 7B. The figure contains the 15 components that make the greatest contribution to the column space mode, while the inset of the figure depicts the entire mode with components shaded according to the different cellular processes to which their roles have been assigned (see the figure legend). The first “eigen-regulatory reaction” (i.e., the first mode of the column space for R*) is spanned by key regulators and their targets. For example, the first mode contains the most ubiquitous transcription factor in *E. coli*, Crp, and the most ubiquitous metabolite in *E. coli*, oxygen, as well as their target genes (as an example, b2799, which expresses FucO, a subunit of L-1,2-propanediol oxidoreductase, is the target gene that contributes most to this mode, as it requires both the absence of oxygen and the presence of Crp for transcription). This result demonstrates that aerobic control is a primary regulatory activity within *E. coli*. Similarly, the second “eigen-regulatory reaction” (the second mode) is spanned by other key regulators, including the absence of ArcA and Fnr as well as their target genes, such as b2284 (*nuoF*, which expresses a subunit of NADH ubiquinone oxidoreductase) (not shown). Therefore, the dominant modes of the column space of R* capture the components of the TRS that systemically affect gene expression.

Another related aspect of the column space is the similarity or dissimilarity of the regulatory reactions driving gene transcription. As described above (see “Methods”), the angle between pairs of columns is indicative of how similarly two gene rules affect the state of the *E. coli* TRS. Figure 7C illustrates the angles between all pair-wise combinations of the columns (in blue) of the regulatory network matrix **R**. There are a total of approximately 14,000 pairings with angles less than 90 degrees. Interestingly, only a few regulatory rules exhibit very small angles (less than 45 degrees), and these are mostly genes that have multiple OR clauses around a particular regulator. Instead, most of the gene rules in the *E. coli* TRS are very different from one another. The relatively few instances of small angels support the hypothesis proposed above that, while operons and regulons are observed within the *E. coli* TRN, control of the network is significantly more distributed than Figure 1B would imply.

#### Left null space

The left null space spans the final *m*−*r* columns or left singular vectors contained in **U** [32]. The first mode of the left null space for R* (containing a randomly-generated environment) is illustrated in Figure 7D. Here the nodes are labeled and shaded according to the different cellular processes in which they participate. In this case, because R* is nearly full rank, only 13 components appear in the left null space, and all of these are extracellular metabolites. In contrast to the column space, the left null space is spanned by the extracellular metabolites that affect few regulatory reactions, such as maltotetraose. Consequently, the left null space contains disconnected components of the system. These features of the network may constitute the most poorly characterized components of the system worthy of further experimental study. Alternatively, they may represent aspects of the *E. coli* TRS that are seldom used but have not yet been selected out of the system through selective pressure in the given environment. Importantly, while we previously described an interpretation of the left null space of R* (see [7]), this particular observation of disconnected system components would not have been seen without inspecting a network with the scope of the genome-scale *E. coli* TRS.

#### Row space

The row space is spanned by the first *r* singular vectors or rows of **V** ^T^ [32]. For metabolic systems, the row space has been shown to contain “eigen-connectivities,” or the metabolic reactions participating in driving the conversions contained in the column space (see above) [36]. For example, in the metabolic network of *E. coli*, synthase reactions and ATP-coupled transporters have the highest reaction participations of the first singular vectors of **V** ^T^ [36]. Similarly, the row space of the *E. coli* R* matrix shown in Figure 7E contains the regulatory rules that drive the relationships observed in the column space. For example, the six regulatory reactions that contribute most—and equivalently—to the row space are b4198_AC_2, b4197_AC_2, b4196_AC_2, b4195_AC_2, b4194_AC_2, and b4193_AC_2, all of which require the same regulators, notably the absence of oxygen, presence of Crp, and absence of transcriptional repressor UlaR, for the transcription of the corresponding gene. Interestingly, the corresponding genes are all part of the same operon within *E. coli*: L-xylulose 5-phosphate 4-epimerase (b4198 or *ulaF*), L-xylulose 5-phosphate 3-epimerase (b4197 or *ulaE*), 3-keto-L-gulonate 6-phosphate decarboxylase (b4196 or *ulaD*), *ulaC* (b4195), *ulaB* (b4194), and *ulaA* (b4193). Consequently, the row space of R* provides insight into similarly expressed genes, or further understanding of operon and regulon structure within the TRN. Importantly, the row space of R* appears to distinguish between two different operons that might be similarly regulated (i.e., effectively “regulons”): the regulatory reactions b0902_AC_3, b0903_AC_3, and b0904_AC_3 are identical to the regulatory reaction b2492_AC_3. Genes *b0902*, *b0903*, and *b0904* are found in a different location from the gene *b2492* within the *E. coli* genome. However, they are all involved in formate transport and constitute a putative regulon identified by this subspace analysis.

In addition, as with the column space, we evaluated the angles between all pair-wise combinations of rows (i.e., network components, including extracellular cues, transcription factors, and target gene products). The results are presented in red in Figure 7C. There are a total of approximately 200 row pairings with angles less than 90 degrees, suggesting that the majority of the components are regulators with unique sets of targets. This finding indicates that the majority of regulators not only affect few genes but also the amount of redundancy in the gene rules within the network is minimal.

#### Null space

The null space of R* is spanned by the *n*−*r* remaining expression states of the *E. coli* TRS (see Figure 7F). As with the row space, these are further “eigen-connectivities” for the system. For example, the first mode demonstrates the activation of a subunit of the RNA polymerase sigma factor (*rpoS*) through RpoS_AC_1. Importantly, the similarities between the row and null spaces of R* in that they both define eigen-connectivities (i.e., expression states) for a TRS emerged through this first-ever genome-scale implementation of the **R** matrix formalism.

## Discussion

The results presented here represent the first steps toward applying the regulatory network matrix formalism at the genome scale. Specifically, we constructed a regulatory network matrix **R** for the genome-scale *E. coli* transcriptional regulatory network, including direct interactions between environmental stimuli, transcription factors, and other downstream target genes. Ultimately, we (1) identified features of the *E. coli* TRN, including the numbers of components and regulatory relationships; (2) validated our model in the context of available experimental data and illustrated how the **R** matrix at genome scale affords predictions of expression states for all possible systemic environments; and (3) characterized the fundamental subspaces of the regulatory system matrix R* for the *E. coli* TRS, noting unique properties about these subspaces of R* not previously observed, including the distributed (and non-hierarchical) nature of the *functional* states of the genome-scale transcriptional regulatory network which is in contrast to that observed for genome-scale metabolic networks.

As illustrated in Figures 1 and 4, for a system of 776 total environmental stimuli, transcription factors, and target genes, **R** scales to 1009 components (rows)×1685 regulatory reactions (columns), including 579 exchange reactions. When coupled with an environment matrix **E**, the size of the eventual R* matrix representing the complete TRS was 1009 rows by 2461 columns. It is reasonable to expect that similar observations will be made for systems that maintain similar distributions of inputs per regulated gene (see Figure 4) as well as multi-subunit complex and isozyme composition for metabolic enzymes and transporters.

Recently, the functional states of a prototypic TRS as well as a small-scale *E. coli lac* operon TRS, as represented by this pseudo-stoichiometric regulatory network matrix formalism, were characterized. The sheer number of environmental stimuli defined in this system, however, prohibits a comprehensive analysis encompassing all possible combinations as was performed for the prototypic TRN in [7]. Instead, we performed a random sampling of all possible environments to characterize key properties of the functional states of the *E. coli* TRS. Specifically, we computed the percentage of these randomly-simulated environments in which the 629 regulated genes were expressed, identifying those genes most significant to the *E. coli* regulatory program as those ubiquitously expressed across the environments. Importantly, this type of *in silico* expression analysis offers an efficient way to characterize differences in a regulatory program across multiple environments. Indeed, our results for two environments for which microarray profiling has previously been completed exhibited strong concordance with the experimental data.

This work constitutes the first genome-scale analysis of the fundamental subspaces of R*, and understanding of these subspaces has been significantly enhanced with the genome-scale implementation. Specifically, we describe how the column and left null spaces of R* are spanned by components of the system that are either very connected or very disconnected, respectively, among the regulatory relationships. For example, the column space of a representative *E. coli* R* matrix contained Crp and oxygen, two key systemic regulators. By contrast, the left null space of a representative matrix contained such extracellular cues as D-galactarate, which are minimally involved in the *E. coli* regulatory program. Thus, the left null space can identify network features that are poorly characterized (and require further experimental interrogation) or network function with minimal effect on phenotype. We further describe how the row and null spaces of R* together describe all possible expression states of the *E. coli* TRS for a given environment.

Interestingly, the singular value spectrum for the *E. coli* TRS is uniformly distributed (see Figure 7A), implying that there are few dominating components or reactions within the system. As described previously, this result contrasts with the network topology observed in Figure 1B as well as the connectivity distributions shown in Figure 4, and it implies that the functional states of the genome-scale *E. coli* TRS are diffuse. Moreover, the result suggests that transcriptional regulation is inherently different from metabolism, in which the first few principal modes sufficiently recapitulate a significant fraction of the underlying stoichiometric network [36]. Although an important caveat to this result is that metabolism is far better studied than regulation, it is noteworthy that control of many complex systems is distributed, including chemical plants, pharmaceutical manufacturing pipelines, electrical power grids, and sensor networks [37]–[39]. A distributed control system (DCS) is one in which there exist multiple controllers, with one or more of these controllers managing each component or “subsystem.” DCSs facilitate cost savings (as there exist fewer input/output connections), improved scalability (as a central node does not become overburdened as additional components are added), and greater redundancy (as no one node serves as a key hub) [37]. These advantages are critically important in biological systems. For example, there is increasing evidence that control of energy balance is distributed through different parts of the brain [40]. As a TRS constitutes the “control system” for a single living cell, a distributed regulatory network seems a likely choice for cells to evolve toward over time. Indeed, a recent study identified a hidden distributed architecture underlying the scale-free TRN of yeast [41]. Similarly, riboswitches, the structured elements found in 5′ untranslated regions of mRNAs that regulate gene expression by binding to small metabolites, have been shown to exhibit distributed functional effects within a genome [42]. Whereas the structure of metabolic pathways remains constant across multiple environments, our findings suggest that there exist many direct and specific (i.e., one-to-one) relationships between a given environment and the sets of genes that are turned “on” (and, in turn, the fluxes through the metabolic pathways). Thus, the uniform distribution of the singular value spectrum that we have observed implies that, in spite of the operon and regulon structure observed in the network topology, there exists a need for a functional analysis rather than a structural one. Further work exploring this type of a relationship in other organisms may provide interesting insights into evolutionary differences in their regulatory programs.

Among the *E. coli* TRN components, 125 are transcriptional regulators. While a few of these are global regulators like Crp and affect the expression of many genes, the majority of regulators control few targets (see Figures 1 and 4). This architecture of the *E. coli* TRN [43] has been explored extensively in recent years [44]. A drawback to these structural studies is that their direct relevance to the functional state of the cell is often unclear since they rely on inferential associations, e.g., based on the functional annotation of target genes to assign causal relationships to identified network motifs.

The work presented here focuses on the network for which relationships between environmental stimuli and transcription factors are directly ascribed. In so doing, relationships between the environment and transcriptional state can be mapped. Furthermore, the representation of the *E. coli* TRS in Figure 1B illustrates the complexity of the network in terms of the numbers of components and interactions. Tracing the edges to determine which genes are transcribed under different environmental conditions would be a difficult process. Instead, representing these interactions in a structured matrix facilitates the use of linear algebra techniques for characterizing emergent properties of a TRS and generating novel hypotheses of the system.

While this work involves an investigation of a previously constructed model of *E. coli*, this formalism may also prove useful for structuring and analyzing emerging high-throughput data for *E. coli*. For example, ChIP-chip data analyzing the genome-wide binding profiles for several microbial transcriptional regulators, including Crp, Fnr, and Lrp as well as various nucleoid binding proteins and sigma factors have appeared [45]–[47]. These results have suggested that, in spite of the interactions that have been characterized thus far, there remains considerable complexity within the *E. coli* TRS that needs to be further elucidated [46]. For instance, as shown in Figure 5C, certain GO categories of genes exhibited much poorer validation than others, suggesting that specific parts of the TRS require further study. The model performed well for aspects of *E. coli* biology that have been thoroughly studied to date, namely regulation of genes involved central metabolism and carbon uptake [27]. By contrast, energy metabolism and building block biosynthesis-related genes exhibited less than 70 percent validation. These results will likely be similar for other organisms as well, as the initial focus of study for biological systems has primarily been metabolism. Importantly, given the distributed functional nature of the *E. coli* TRS, the probability of a single incorrect gene expression prediction resulting in a large-scale reduction in accuracy (owing to residual effects upon downstream target genes) is small. Specific genes whose model-predicted expression states did not match with experimental measurements will nevertheless need to be explored. A defined environmental perturbation is critical for proper mapping of regulatory response and interactions with downstream targets. Furthermore, ChIP-chip data in isolation are not sufficient for this methodology to be successful. Corresponding transcriptional profiling data in order to derive directionality of regulation (i.e. up or downregulation of targets) are also important. Relatively conservative criteria should be used in incorporating these data into the **R** matrix.

Importantly, in spite of the advances using a **R** matrix formalism, there are certain limitations to the pseudo-stoichiometric approach that we have utilized for representing the *E. coli* TRS. In particular, as our reconstruction is based on an existing Boolean model, it is binary both at the level of control (i.e., how inputs affect individual genes) and expression (i.e., genes are predicted to be turned “on” or “off” in response to a given environment). In the future, mechanisms for incorporating species concentrations (particularly at the level of inputs) will need to be incorporated. In addition, during our analysis, the dynamics of the TRS are approximated. Although for a given environment we compute the sequence of expression states before a “steady-state” (or oscillation) is observed effectively tracing through the dynamics, we do not incorporate time explicitly.

Nevertheless, the pseudo-stoichiometric approach to defining regulatory interactions is akin to existing stoichiometric strategies for modeling metabolic and signaling systems. Accordingly, future work could directly incorporate the regulatory equations described herein to develop a comprehensive model of the cell [48]. For example, a recent approach characterizing dynamic properties of integrated signaling, metabolic, and regulatory systems is predicated on the availability of stoichiometric or pseudo-stoichiometric matrix representations of these types of systems [49]. Ultimately, in this way, the application of the R* matrix to genome-scale regulatory networks may enable the quantitative investigation of emergent properties of biochemical systems, including whole-cell dynamics.

## Supporting Information

Dataset S1 The genome-scale *Escherichia coli* transcriptional regulatory system. This list provides the genes and regulatory rules that are contained within our model of genome-scale *E. coli* transcriptional regulation.
(0.09 MB XLS)

Dataset S2 Randomly-sampled environments. The expression states were predicted by our **R** matrix approach for each of these 1000 randomly-sampled environments.
(0.96 MB XLS)

Dataset S3 Anaerobic and aerobic minimal media. These anaerobic and aerobic minimal media conditions (reported in [13]) were used to validate our **R** matrix representation of the *E. coli* transcriptional regulatory system.
(0.04 MB XLS)

Dataset S4 Gene legend for Figure 5A. A legend that defines the genes as they are presented in the differential gene expression diagrams in Figure 5A is provided.
(0.03 MB XLS)

Dataset S5 Interesting genes identified from expression states predicted for randomly-sampled environments. Figure 6A illustrates (rank-ordered) the percentage of the 1000 randomly-sampled environments in which the 629 regulated genes within the **R** matrix model are expressed. Genes that were always expressed, never expressed, or expressed in about 50 percent of the randomly-simulated environments are listed here.
(0.21 MB XLS)

Dataset S6 Details about the clusters contained in Figure S1. The gene expression correlation matrix was clustered, using standard hierarchical clustering techniques described in Text S3 and as shown in the resultant dendrogram in Figure S1. Details about the clusters highlighted in Figure S1 are presented.
(0.14 MB XLS)

Protocol S1 Expression parser. For each gene, this Perl script converts Boolean statements into regulatory reactions (i.e., pseudo-stoichiometric relationships) that may be represented in a regulatory network matrix (**R**).
(<0.01 MB ZIP)

Protocol S2 MATLAB implementation of the genome-scale *E. coli* **R** and R* matrices. The **R** matrix and a sample R* matrix are represented as variables within a MATLAB workspace. See the comments within the accompanying MATLAB m-file for details.
(0.07 MB ZIP)

Text S1 Alternate objective functions for computing expression states. As part of our linear programming (LP) framework for computing expression states, we considered several different objective functions. These objective functions and the corresponding results are summarized.
(0.01 MB PDF)

Text S2 Singular value decomposition (SVD) for exploring fundamental subspaces of R*. Singular value decomposition (SVD) is described in the context of its application to the R* matrix.
(0.11 MB PDF)

Text S3 Clustering the gene expression correlation matrix. The gene expression correlation matrix was clustered, using standard hierarchical clustering techniques. The clustering methodology and results are presented (see Figure S1 and Dataset S6 as well).
(0.01 MB PDF)

Figure S1 A dendrogram of the clustered gene expression correlation matrix. The gene expression correlation matrix was clustered, using standard hierarchical clustering techniques described in Text S3. The resultant dendrogram is presented.
(0.56 MB EPS)
