# Supplementary material for: Functional States of the Genome-Scale Escherichia Coli Transcriptional Regulatory System
Source: PLoS Comput Biol. 2009 Jun 5;5(6):e1000403. doi: 10.1371/journal.pcbi.1000403 (PMC2685017; doi:10.1371/journal.pcbi.1000403)
Supplement: Text S1 — Alternate objective functions for computing expression states. As part of our linear programming (LP) framework for computing expression states, we considered several different objective functions. These objective functions and the corresponding results are summarized. (0.01 MB PDF) [file pcbi.1000403.s009.pdf]

Functional states of the genome-scale *Escherichia coli* transcriptional regulatory system

By Erwin P. Gianchandani, Andrew R. Joyce, Bernhard Ø. Palsson, and Jason A. Papin

Text S1: Alternate objective functions for computing expression states

As described in the manuscript, we used a linear programming (LP) strategy to efficiently predict a route through the transcriptional regulatory network (TRN) given an environment, i.e., an expression profile or state (see Figure 3A of the manuscript). Our LP strategy involves iterating through the regulatory reactions contained within the transcriptional regulatory system (TRS) matrix **R***, optimizing the "flux" associated with each reaction (line 5 in Figure 3A). If we observed that we were able to obtain a flux distribution with a nonzero flux through the "optimized" reaction, then we predicted that the corresponding reaction is "active" and associated gene "expressed." This process of optimizing for the flux through each regulatory reaction was repeated multiple times until the gene expression predictions (i.e., "expressed" or "not expressed") were consistent for all the genes within the model across multiple iterations (i.e., a "steady-state" was achieved) or until an oscillation characteristic of one or more feedback loops was observed in the regulatory relationships. Complete details of our LP strategy are presented in the manuscript.

Importantly, we evaluated different objective functions as part of our LP problem formulation. However, only the strategy described above (and in the manuscript) yielded valid results. For example, we considered simultaneously optimizing for all the regulatory reactions corresponding to a given gene (as opposed to each reaction independently), and repeating this process for all the genes within the *E. coli* TRN. We also optimized all the regulatory reactions (spanning all the genes) within the TRN concurrently. In both cases, the results (i.e., the expression profiles corresponding to a given environment) were incorrect when contrasted with what one would expect from simple inspection of the underlying Boolean rules. Ultimately, the LP strategy that we used is predicated on a direct, one-to-one balance between a given regulatory reaction, the inputs that are required for the reaction to be "active," and the corresponding output (i.e., whether the gene can be transcribed). Furthermore, the matrix-based formalism offers efficient computation of the expression state.
