# Supplementary material for: Functional States of the Genome-Scale Escherichia Coli Transcriptional Regulatory System
Source: PLoS Comput Biol. 2009 Jun 5;5(6):e1000403. doi: 10.1371/journal.pcbi.1000403 (PMC2685017; doi:10.1371/journal.pcbi.1000403)
Supplement: Text S2 — Singular value decomposition (SVD) for exploring fundamental subspaces of R*. Singular value decomposition (SVD) is described in the context of its application to the R* matrix. (0.11 MB PDF) [file pcbi.1000403.s010.pdf]

Functional states of the genome-scale *Escherichia coli* transcriptional regulatory system

By Erwin P. Gianchandani, Andrew R. Joyce, Bernhard Ø. Palsson, and Jason A. Papin

Text S2: Singular value decomposition (SVD) for exploring fundamental subspaces of R*

Singular value decomposition (SVD) is used often to analyze properties of matrices and the datasets that they represent (see [1], [2], and [3] for examples). SVD decomposes a matrix into three matrices, often named **U**, **Σ**, and **V** (see Figure 3B) [1], and these matrices delineate the four fundamental subspaces of the original matrix (see [4] and Figure 4B). We performed SVD on each **R***, as described in [4] and shown in Figure 3B, yielding $\mathbf{R^*} = \mathbf{U} \bullet \mathbf{\Sigma} \bullet \mathbf{V}^T$. The diagonal entries of the matrix $\mathbf{\Sigma} = \mathrm{diag}(\sigma_1, \sigma_2, \ldots, \sigma_r)$, where r is the rank of **R*** and $\sigma_1 \geq \sigma_2 \geq \ldots \geq \sigma_r$, indicate the relative contribution of the corresponding left singular vector (a column of **U**) and right singular vector (a row of $\mathbf{V}^T$) in the overall construction of the TRS [3]. Note that an important feature of SVD is that the singular vectors are orthonormal to each other and consequently each principal mode is decoupled from all the others.

Briefly, we previously reported that the null space of the **R*** matrix contains the collection of all possible vectors describing active/inactive genes (columns of the matrix) that balance the TRN with the environmental cues [4]. In other words, the null space of **R*** captures all possible balanced expression (i.e., functional) states of the TRS that it represents. The left null space of **R*** contains pools or aggregates of network components that are invariant across all the regulatory rules of the TRS. Thus, these pools represent node-neutral states of the TRS. These pools may be groups of open reading frames that are coordinately regulated and can be classified as regulated units, or regulons. As we describe in the "Results" section of the manuscript, our understanding of the four fundamental subspaces of **R***, which we originally proposed in [4] on the basis of our work with two small-scale systems, has been considerably enhanced by the extension of **R** and **R*** to the genome-scale *E. coli* TRS.

References

1. Lay DC (2006) Linear algebra and it's applications. Boston: Pearson/Addison-Wesley. 1 v. (various pagings) p.
2. Alter O, Brown PO, Botstein D (2000) Singular value decomposition for genome-wide expression data processing and modeling. Proc Natl Acad Sci U S A 97: 10101-10106.
3. Famili I, Palsson BO (2003) Systemic metabolic reactions are obtained by singular value decomposition of genome-scale stoichiometric matrices. J Theor Biol 224: 87-96.
4. Gianchandani EP, Papin JA, Price ND, Joyce AR, Palsson BO (2006) Matrix formalism to describe functional states of transcriptional regulatory systems. PLoS Comput Biol 2: e101.
