# Supplementary material for: Functional States of the Genome-Scale Escherichia Coli Transcriptional Regulatory System
Source: PLoS Comput Biol. 2009 Jun 5;5(6):e1000403. doi: 10.1371/journal.pcbi.1000403 (PMC2685017; doi:10.1371/journal.pcbi.1000403)
Supplement: Text S3 — Clustering the gene expression correlation matrix. The gene expression correlation matrix was clustered, using standard hierarchical clustering techniques. The clustering methodology and results are presented (see Figure S1 and Dataset S6 as well). (0.01 MB PDF) [file pcbi.1000403.s011.pdf]

Functional states of the genome-scale *Escherichia coli* transcriptional regulatory system

By Erwin P. Gianchandani, Andrew R. Joyce, Bernhard Ø. Palsson, and Jason A. Papin

Text S3: Clustering the gene expression correlation matrix

As described in the manuscript, we generated a gene expression correlation matrix (shown in Figure 6B) containing the level of expression correlation across the 1000 randomly-simulated environments for every pair of genes within the *Escherichia coli* transcriptional regulatory system (TRS). Once we obtained this expression correlation matrix, the matrix was clustered into groups of genes with similar correlation profiles (see Figure S1). Standard hierarchical clustering techniques were employed using MATLAB v. 7.6 (part of the R2008a release package, MathWorks, Inc., Natick, MA), including a Euclidean distance metric along both the rows and columns of the expression correlation matrix. Note that, unlike in Figure 6B in the manuscript, symmetric values (i.e., values above and below the diagonal) were included during clustering. The bands at the top of Figure S1 correspond to groups of genes whose expression correlation patterns are similar, i.e., the resultant clusters.

The genes within each cluster were associated with gene ontology (GO) categories, and the numbers of genes within each cluster corresponding to each GO category were tabulated (see Dataset S6). In general, for each cluster, a majority of genes belonged to a single GO category, further suggesting that *E. coli* is able to induce direct, specific responses to a given environment.

In addition, interestingly, the majority of the clusters contained genes whose regulatory rules were independently validated (per the data for the anaerobic-aerobic minimal media shift reported in the manuscript) (see Dataset S6). Of the 12 clusters that were selected for further analysis, seven exhibited > 73 percent validation, i.e., they exceeded the level of validation that was observed for the complete genome-scale model. By contrast, two clusters exhibited exceptionally poor validation, with just 6 and 10 percent of the genes contained within these clusters being correctly predicted by our model (in the case of a shift from anerobic to aerobic minimal media). These results suggest that “correlated gene sets” contain genes whose regulation has been similarly characterized.

These clustered correlation profiles serve to further emphasize the novel insights about structural and functional properties of the system that may be hypothesized via the regulatory network matrix formalism and associated analysis.
